# The Polymers of Life: Exploring Cellular Function Through Polymer Concepts

**DOI:** 10.1002/advs.75240

**Published:** 2026-05-19

**Authors:** Mark Chen, Ashutosh Chilkoti

**Affiliations:** ^1^ Department of Radiation Oncology Duke University Durham NC USA; ^2^ Department of Biomedical Engineering Duke University Durham NC USA

**Keywords:** biomolecular condensates, gene regulation, phase separation, polymer physics, synthetic biology

## Abstract

Biological organization has traditionally been viewed through the lens of distinct organelles and lock‐and‐key molecular interactions. The recent explosion of interest in phase separation has reshaped this view, revealing that cells also organize through membraneless organelles, also called biomolecular condensates, for spatial, temporal, and functional organization of its constituents. A key outcome of this shift has been the integration of polymer physics into cell biology, opening new avenues for understanding macromolecular behavior in living systems. This review traces the evolution of the field from foundational polymer physics, connects polymer properties to biological processes, and proposes a framework for interrogating cellular function through the lens of polymer science. While polymers are ubiquitous in daily life, their relevance to natural macromolecules and biological organization in cells is less apparent outside the discipline. Notably, current explorations at the biology–polymer interface echo much of the early pioneering work in polymer science. Accordingly, this review serves both as a primer on polymer concepts central to cellular processes and as a synthesis of recent advances and emerging tools for studying condensates in living cells.

## Graphical abstract

Biomolecular phase separation reveals that a hidden layer of cellular organization is governed by the principles of polymer science. This review bridges polymer physics and cell biology, offering a primer on fundamental concepts, proposing a framework for interrogating cellular function, and synthesizing biophysical methods for decoding macromolecular behavior and the fundamental polymers of life.

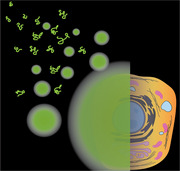

## 1 Introduction to Foundational Polymer Concepts

For much of the 20th century, cellular organization was understood primarily through structural hierarchy and binding specificity. Following Emil Fischer's late 19th century “lock‐and‐key” model, biological function was largely conceived as a series of deterministic events: a ligand binds a receptor, an enzyme binds a substrate, and a transcription factor binds a promoter [1]. This biochemical perspective, while highly successful in mapping metabolic pathways and gene regulatory networks, often portrayed biological macromolecules as isolated entities operating in a dilute, aqueous environment—an image reinforced by textbook depictions [2]. Yet this framework overlooked the crowded, viscous, and thermally noisy environment of the cellular interior, where discrete models captured only fragments of a far more complex picture.

The recent shift toward “physical biology” represents a paradigm shift, comparable to the birth of physical chemistry in the late 19th century. Biological macromolecules are now recognized not as static structures, but as dynamic polymers governed by the laws of soft matter physics. Within a cell, macromolecular concentrations can exceed 400 mg/mL creating conditions vastly different from the dilute conditions of test tubes [3, 4]. Molecular crowding and excluded volume effects dominate intermolecular interactions in the cell [5, 6]. Understanding cellular processes therefore requires moving beyond cataloging molecular components to characterizing their dynamic behaviors. In this review, we introduce foundational concepts from polymer science to encourage nonspecialists to approach cellular processes through the lens of polymer physics.

**FIGURE 1:**
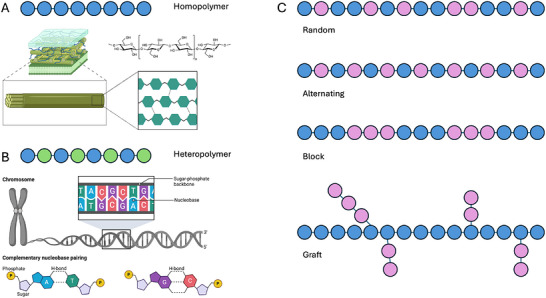
Overview of polymer structures found in biology. (A) Homopolymers consist of one type of monomeric repeat as found in cellulose, a polymer of β‐d‐Glucose. (B) Heteropolymers, or copolymers, consist of two or more types of monomeric units as found in DNA, a polymer of four different nucleotides. Some regions of DNA may act as homopolymers, such as the poly‐A tail. (C) Different types of copolymers. Random copolymers are comprised of randomly arranged monomer units (A–B–A–B–B–A–B–A–A). Alternating copolymers consist of alternating monomer units (A–B–A–B–A–B). Block copolymers consist of alternating homopolymer blocks (A–A–B–B–A–A–B–B). Graft copolymers consist of chains of homopolymers. Partially created in BioRender: Chen, M. (2026), https://BioRender.com/tq73zeg.

Polymers are molecules composed of long covalently linked sequences of monomers (Figure 1). These chains whether linear, branched, or networks, underpin the diverse properties of both synthetic and natural polymers. Homopolymers consist of repeated units of a single monomer, represented as‐[A]*_n_*‐, where *n* is the number of repeat units (Figure 1A). In contrast, copolymers incorporate multiple monomers in arrangements that can be random, alternating, block, or graft (Figure 1B,C). Although these distinctions may seem elementary today, the concept of macromolecules was not obvious until Staudinger demonstrated that materials such as natural rubber were comprised of long atomic chains rather than aggregates held together by weak physical forces [7]. His pioneering work earned him the 1953 Nobel Prize in Chemistry in 1953.

The theoretical foundations of polymer science were largely established by Flory and de Gennes [8, 9]. Originally developed to describe rubber and synthetic polymers, their frameworks have proven unexpectedly relevant to biology [10, 11, 12]. Flory showed that polymer conformation is driven by entropy maximization: coiled states (high entropy) are statistically favored over stretched states (low entropy) [8]. This principle rooted in the second law of thermodynamics explains phenomena such as DNA recoil and fluctuations of intrinsically disordered protein chains [13, 14]. It also reframed biological structure as probabilistic ensembles rather than rigid lock‐and‐key fits.

Building upon Flory's work, de Gennes introduced scaling concepts to predict how polymer properties change with length and concentration [9]. Particularly relevant to cellular biology is de Gennes’ theory of reptation, which describes the snake‐like motion of a polymer chain constrained within a network of other chains. In the entangled environment of the cell nucleus or the cytoskeletal network, large biopolymers cannot move freely in three dimensions and must slide along their own contours like a snake [11]. This concept has become indispensable for modeling genome reorganization and mRNA transport in the dense cellular milieu [15, 16, 17]. For this work, he was awarded the 1991 Nobel Prize in Physics.

Another cornerstone was laid by Tanaka, who experimentally demonstrated that polymer networks undergo dynamic phase transitions. His discovery of volume phase transition revealed that polymer gels can collapse or swell dramatically in response to minute environmental changes such as temperature, pH, or solvent composition [18, 19]. These behaviors provided a physical basis for understanding how biological polymers act as sensitive switches, converting subtle chemical signals into mechanical responses [20, 21, 22, 23, 24, 25].

**FIGURE 2:**
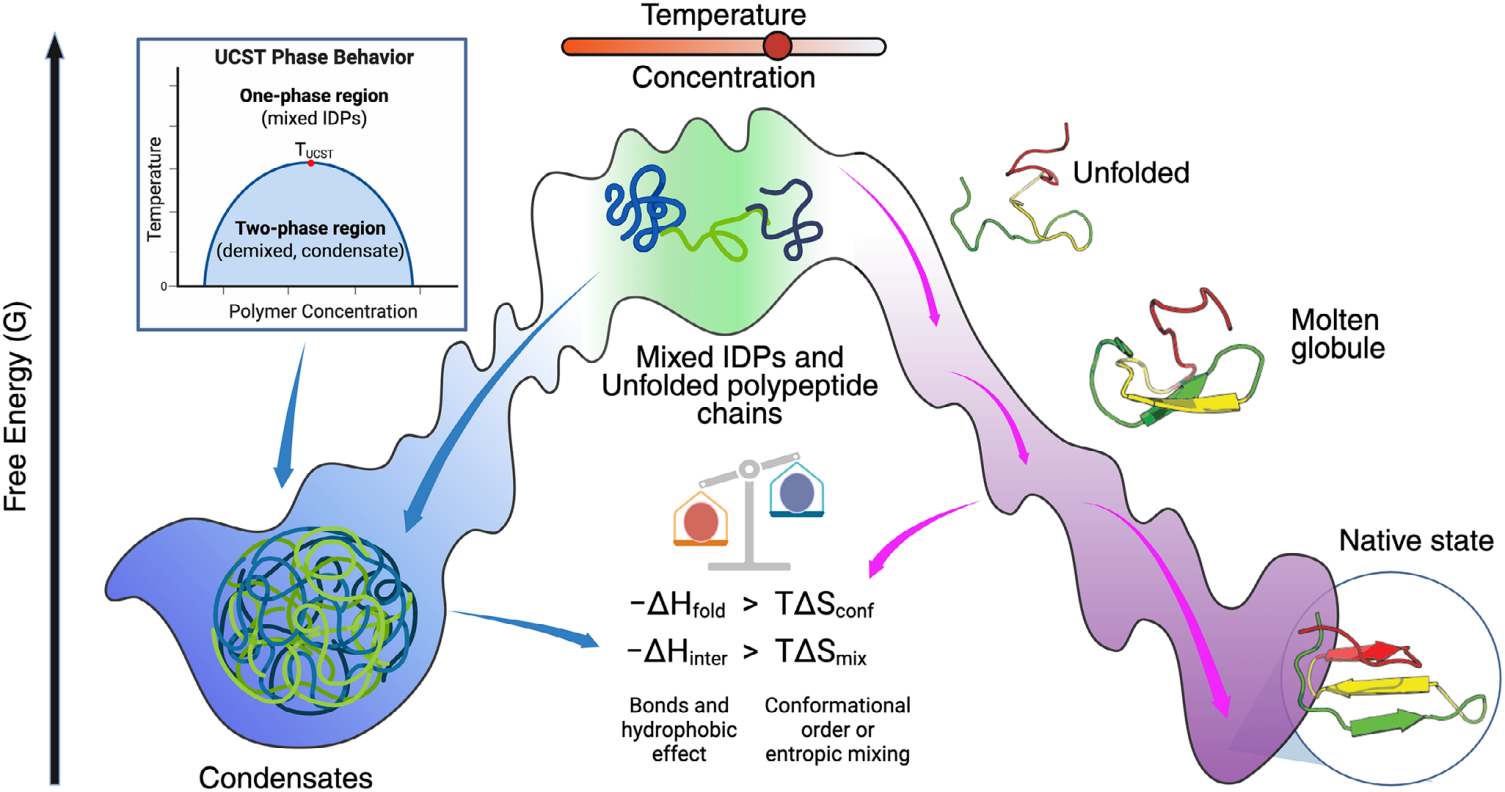
Tug‐of‐war between entropy and enthalpy. Similar to unfolded polypeptide chains, a mixture of intrinsically disordered proteins (IDPs) has high conformational entropy (disorder). Upper critical solution temperature (UCST) behavior is an example where condensation is the result of a loss of randomness in the protein, driven by self‐association and largely hydrophobic collapse. At *T* > *T*_UCST_, the entropic mixing (*T*Δ*S*_mix_ dominates and the landscape is homogeneous with high entropy (disordered IDPs). At *T* < *T*_UCST_, the enthalpic interactions (Δ*H*_inter_) between chains predominate leading to phase separation. For folded proteins, −Δ*H*_fold_ competes with *T*Δ*S*_conf_ (cost of conformational order) to drive folding. Protein/condensate stability can be disrupted/unfolded with the addition of energy such as heat to favor entropy. Created in BioRender: Chen, M. (2026), https://BioRender.com/tq73zeg.

Today, cellular function is increasingly recognized as an emergent property of polymer dynamics [26]. Activity depends not only on the presence of biomolecules, but also on their phase behavior, topological constraints, and viscoelastic properties [27, 28, 29, 30]. For example, liquid–liquid phase separation (LLPS), predicted by the Flory–Huggins solution theory, enables the formation of membraneless organelles (biomolecular condensates) that segregate macromolecules within a cell [31, 32]. This theory describes the balance between entropy, favoring mixing, and enthalpy, favoring self‐association (Figure 2). When enthalpic forces between polymer chains dominate over polymer–solvent interactions, polymers phase separate and form condensates. Condensates can also form via entropic driving forces when polymer–solvent interactions are disfavored: lower entropy bound water molecules return to higher entropy bulk water, providing the gain in entropy required to drive phase separation. Beyond compartmentalization, polymer physics also governs cellular mechanics, where cytoskeletal cross‐linking provides the rigidity essential for tissue integrity [33, 34].

This review seeks to bridge cell biology and polymer physics, demonstrating how physical principles underpin biological function. We highlight experimental and theoretical insights into polymer dynamics, introduce emerging concepts, and discuss open questions at the interface of polymer science and cellular function. By reimagining cell biology through the lens of polymer physics, we aim to foster a more mechanistic and predictive understanding of life at the molecular level.

## 2 Classes of Biopolymers

The eukaryotic cell contains three major classes of biopolymers: polynucleotides (DNA, RNA), polypeptides (proteins), and polysaccharides (cellulose, glycogen, etc.). Here we review polymer concepts applicable to each of these classes that are essential to the central dogma of life. Specialized classes of biopolymers found in specific organisms like bacteria or certain plants will also be briefly discussed. Lipids are not classified as a biopolymer class because they are not formed by repeating chains of covalently bonded monomers.

### 2.1 Polynucleotides: Polymers of Information Storage and Transfer

#### 2.1.1 DNA and the Packing Problem

DNA is a linear polymer that was once considered too simple to encode complex genetic information. It also presents one of biology's most striking storage challenges: packing of the genome (Figure 1B). In humans approximately 2 m of DNA must be compacted ∼200,000‐fold into a nucleus only 5–10 µm in diameter [35]. If DNA behaved as a simple equilibrium polymer, it would collapse into an equilibrium globule in its compacted state. An equilibrium globule forms when only physical forces act on the DNA and it becomes deeply entangled forming a globule full of “knots,” which cannot be untangled when compacted into the nucleus. However, such a state would be incompatible with life because it would prevent strand accessibility required for transcription and replication [16, 36]. Instead, cells employ a hierarchical folding strategy mediated by architectural proteins like histones, producing a fractal (or crumpled) globule—a concept initially proposed by Grosberg [37]. At the lowest level, DNA wraps around histone octamers to form a 10 nm fiber, reducing its contour length [38]. Higher‐order chromatin organization has long been a subject of debate, but recent advances using chromosome conformation capture (Hi‐C) have provided strong evidence that the genome adopts a fractal globule state [36, 39, 40]. Unlike the knotted equilibrium globule, the fractal globule is a polymer state formed by the rapid collapse of a chain into a compact, but unknotted conformation. Lieberman–Aiden et al. demonstrated that human chromosomes fold into this fractal state, which preserves the local ordering of the linear sequence in 3D space [36].

The functional consequence of this architecture is its accessibility: because the polymer is unknotted, specific loci can be readily extruded to the surface of the globule for transcription and then retracted, much like pulling a thread from an untangled skein of yarn. This “crumpled” state enables extreme compaction while avoiding the topological deadlock of reptation‐limited entanglement. Supporting this model, asymmetric cohesion‐induced loop extrusion has been directly visualized in real‐time in yeast and demonstrates how structural maintenance of chromosomes complexes restructure genomes [41]. Yet DNA loop extrusion alone is not sufficient for genome reorganization. Rather, it operates in concert with biomolecular condensates, which provide an additional layer of structural and functional regulation [42].

#### 2.1.2 Phase Separation and Chromatin Organization

Beyond topology, the spatial distribution of DNA is strongly influenced by phase separation. The nucleus is partitioned into transcriptionally active euchromatin and inactive heterochromatin. While these compartments were traditionally explained through biochemical modifications such as methylation, acetylation, and phosphorylation, recent evidence suggests they also arise from polymer–polymer incompatibility [43]. Strom and colleagues demonstrated that heterochromatin domains form via liquid–liquid phase separation, driven by the multivalent interactions of heterochromatin protein 1 (HP1). In this model, phase separation organizes the DNA/nucleosome polymer into dense, liquid‐like domains, that sequester chromatin from the transcriptional machinery, thereby silencing gene expression.

Conversely, phase separation can also promote transcriptional activation as shown for the YAP gene and other examples [28, 44, 45]. Gene regulation thus emerges, at least in part, from chromatin organization driven by polymer solubility. By modulating the solvent quality of the nucleoplasm or the interaction strength of chromatin‐binding proteins, cells can induce condensation or dissolution of nuclear compartments. These processes are regulated by mechanisms that remain incompletely understood, though post‐translational modifications (PTMs) and other polymers, including RNA, are known to play critical roles [46, 47, 48, 49, 50].

#### 2.1.3 RNA as a Polymer Scaffold

Unlike genomic DNA, which is predominantly double‐stranded, RNA is single‐stranded, and more flexible and capable of folding into complex secondary and tertiary structures [51]. From a polymer standpoint, RNA behaves more like a branched polymer than a linear chain [52, 53]. Its folding reflects a balance between maximizing base‐pairing enthalpy and minimizing the entropic penalty of forming loops, producing a diverse landscape of hairpins, loops, and pseudoknots that dictate its hydrodynamic radius and interaction surface [51, 54, 55].

The classical lock‐and‐key model casts RNA is a passive messenger, shuttling information from the nucleus to the cytoplasm for translation. By modulating phase separation RNA can influence both the rate of gene expression and composition of transcriptional hubs [56, 57, 58, 59, 60]. A particularly important emerging role is its function as a scaffold for intracellular compartmentalization. Many membraneless organelles—including nucleoli, stress granules, and paraspeckles—are actually ribonucleoprotein (RNP) bodies composed of both RNA and protein complexes [61, 62, 63, 64, 65]. RNA lowers the energetic threshold for phase separation in these assemblies [50, 59, 66], largely due to multivalency—the ability of a single RNA molecule to bind multiple proteins simultaneously. Long noncoding RNAs (lncRNAs) act as extended, flexible tethers that recruit specific RNA‐binding proteins, increasing the local concentration of interacting domains [62, 67, 68]. Studies of RNP dynamics show that RNA sequence and secondary structure can tune the viscosity and material properties of the condensate. By serving as seeds for nucleation, RNA and its associated regulatory proteins help ensure that phase separation occurs at the correct place and time within the cell [56, 69, 70].

### 2.2 Polypeptides: Polymers of Molecular Machinery and Assembly

#### 2.2.1 The Protein Order–Disorder Spectrum

Protein function has traditionally been viewed through the lens of a polypeptide folding into stable, 3D structures [71]. This process was driven by hydrophobic collapse of the sequence and formation of secondary bonds between residues, such as H‐bonding, electrostatic interactions, and disulfide bridges.

**FIGURE 3:**
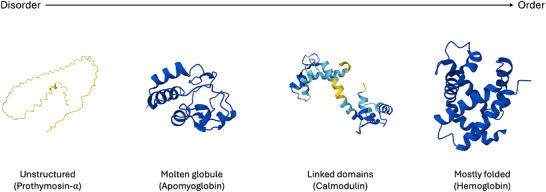
The order—disorder spectrum of protein structures. Completely unstructured proteins such as Prothymosin alpha lack a fixed secondary structure at physiologic pH (P06454). Molten globules such as cytochrome c have secondary structure but lack tight tertiary packing in the absence of a cofactor (heme) (P99999). Proteins such as calmodulin with modular function are often folded domains connected by disordered linkers (P0DP23). Hemoglobin is a mostly folded protein with a stable folded core (alpha subunit shown, Q90487). Structures were sourced from the AlphaFold Protein Structure Database. Model confidence ranges from very low (orange, pLLDT < 50) to low (yellow, pLLDT > 50) to high (light blue, pLLDT > 70) to very high (dark blue, pLLDT > 90). pLDDT is a measure of per‐residue confidence score based on the local distance difference test (lDDT).

It is now increasingly recognized that protein structure spans a continuum from highly ordered to largely disordered states (Figure 3). A substantial fraction of the eukaryotic proteome (over 30%) is composed of intrinsically disordered proteins (IDPs), which contain both folded domains and intrinsically disordered regions (IDRs) [71, 72, 73]. IDRs are notably enriched in disease‐associated mutations, yet these regions have historically received far less attention than structured domains [74, 75]. Early work in the 2000s challenged the assumption that disorder equates to a lack of function, arguing instead that disorder encodes specific and biologically important functions in proteins [71]. These include the capacity to undergo phase transitions and disorder‐to‐order transitions, processes that are highly relevant to biology and disease (Figure 3) [74, 76, 77, 78, 79].

#### 2.2.2 IDPs Driving Phase Separation

**FIGURE 4:**
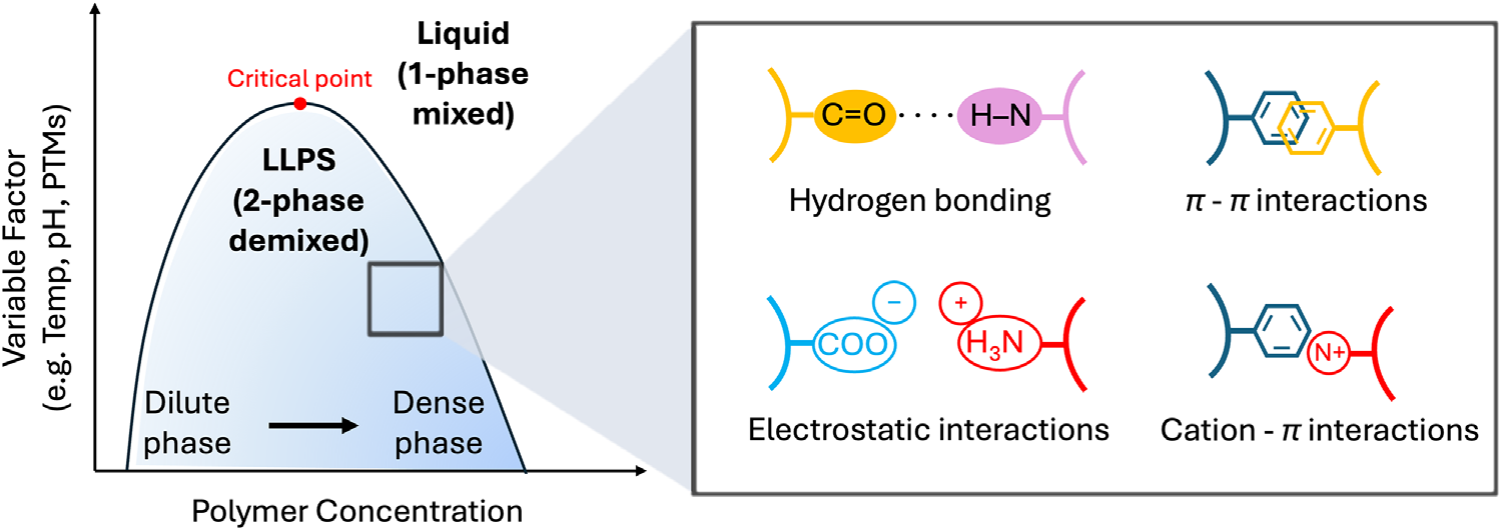
Phase diagram of LLPS and interactions driving a stickers‐and‐spacers model. A schematic representation of the binodal curve separating a soluble single‐phase liquid region from the two‐phase demixed region (blue gradient). An upper critical solution temperature (UCST) curve is depicted, which defines the boundary below which LLPS occurs. The critical point represents the conditions where the two phases have identical composition. Another form of LLPS is lower critical solution temperature (LCST) behavior, which unlike UCST is driven by entropy and LLPS occurs above the phase boundary (not depicted). The stickers‐and‐spacers model is a concept where flexible chains are composed of stickers and spacers, which driving the assembly and formation of a condensate. Key molecular interactions between amino acids of an IDP/IDR that function as the primary stickers in these proteins include hydrogen bonding, electrostatic interactions, and π–π stacking interactions between aromatic rings.

From a polymer physics standpoint, IDPs can be modeled as flexible polymers in a theta solvent, in which polymer–polymer and polymer–solvent interactions are perfectly balanced, allowing them to rapidly sample a vast ensemble of conformations that reveal local structural preferences [80, 81]. These properties confer molecular specificity and enable IDPs to act as springs, linkers, and scavengers that sequester biomolecules. Their polymeric nature also underlies their central role in intracellular phase separation. IDPs are typically enriched in specific amino acids (such as glycine, serine, glutamine, and aromatic residues like tyrosine and phenylalanine) that disfavor stable folding but promote weak, transient interactions (Figure 4). Interactions including π–π stacking between aromatic rings and cation–π interactions between basic residues and aromatic residues allow IDP chains to associate without forming rigid aggregates [82, 83, 84]. When the concentration of these “sticky” polymers exceeds a critical threshold, the system demixes into a protein‐dense phase and a protein‐dilute phase, analogous to oil separating from water.

The recognition that the cytoplasm and nucleoplasm are organized through LLPS represents one of the most transformative shifts in cell biology over the past 15 years. This framework emerged from evidence that cells can establish intracellular compartments without lipid membranes by forming structures now known as biomolecular condensates. Although disordered protein regions had been studied for decades, the field's inflection point was the 2009 study by Brangwynne et al. on P‐granules in *C. elegans* embryos [85]. Their observations—that these germline granules fused upon contact, rounded due to surface tension, and flowed under shear—demonstrated that these organelles behave as liquids rather than solid aggregates. Since then, condensates have been identified across a broad range of biological contexts [15, 58, 68, 86, 87, 88, 89, 90]. Despite this rapid expansion, the molecular architecture and functional consequences of condensates remain incompletely understood, underscoring the need for rigorous mechanistic studies and new tools to study the consequences of condensate behavior in vivo [91, 92, 93, 94].

#### 2.2.3 Sticker‐Spacer Interactions and the Regulation of Phase Separation

LLPS provides a robust framework for understanding how cells spatially organize biochemical reactions without relying on membranes. Early models treated IDPs as isotropic colloids—essentially generic spheres—but the field has since shifted toward the “stickers and spacers” model [95, 96, 97]. Derived from associative polymer theory, this model views proteins as heteropolymers whose phase behavior is governed by the precise linear arrangement of the “sticker” residues that drive intermolecular attraction and “spacer” residues that confer solubility and flexibility [98, 99, 100]. Stickers often include short interactive motifs such as aromatic residues or charged blocks, whereas spacers are typically flexible linkers.

A key insight is that sticker interactions are driven by short‐range, anisotropic forces rather than simple hydrophobicity. As a result, the commonly used “oil in water” metaphor for LLPS is not always accurate. Multivalency—the number of associative domains within a single polypeptide chain—is another central concept and a primary driver of phase separation. When valency exceeds a critical threshold, a sharp phase transition occurs as demonstrated by Rosen and colleagues using neural Wiskott–Aldrich syndrome protein (N‐WASP) to reveal a switch‐like transition [101]. In IDPs, stickers correspond to specific amino acids that interact predominantly through cation–π and π–π forces. A comprehensive mutagenesis study of FUS family proteins identified tyrosine and arginine as principal residues driving phase separation [82]. These residues determine the saturation concentration (*C*_sat_) of FUS; for example, increasing arginine content lowers the *C*_sat_. Electrostatic repulsion provides a second layer of regulation by inhibiting self‐association between prion‐like domains. Together, these interactions are tunable through the sequence and composition of the IDRs in IDPs, enabling cells to fine‐tune phase boundaries by modulating the energetic cost of demixing. Spacers complement this regulation by shaping the entropic cost of condensation and influencing LLPS in three additional ways: (1) determining polymer solubility, (2) modulating polymer cooperativity, and (3) defining condensate material properties [82, 96].

A critical realization in recent years is that “liquid” condensates are rarely static. Sequence‐encoded stickers and spacers act as molecular “rheostats” that can be dynamically tuned through PTMs of IDPs. For example, phosphorylation introduces negative charges into the low‐complexity domain (LCD)—repetitive sequences bound in many RNA‐binding proteins such as FUS hnRNPA2, and TDP‐23 [102, 103, 104]—of FUS, disrupting cation‐π interactions required for phase separation [77]. Numerous other PTMs, including ubiquitylation [105, 106], sumoylation [107], methylation [108, 109], acetylation [110, 111], myristylation [112], and PARylation [113, 114] have been shown to modulate condensate properties in increasingly sophisticated ways.

Condensates can also undergo maturation, transitioning from liquid droplets to gels or solids over time. This aging process, often driven by mechanisms such as Ostwald ripening or fibrillization, can be either pathological (as in neurodegeneration) or functional. Although many IDPs are modeled as flexible entropic chains, McKnight's work provided a crucial structural counterbalance. His studies of LCDs revealed that these regions are not purely disordered. Instead, they can adopt defined structural states. For example, the LCD of FUS can polymerize into labile, amyloid‐like fibers stabilized by “cross‐beta” interactions [115]. Unlike pathological amyloids, these assemblies are dynamically reversible, suggesting that cells exploit not only liquid demixing, but also controlled crystallization or gelation to organize intracellular matter. McKnight's findings highlight a structural polymer physics perspective in which the formation of weak, periodic cross‐links create adaptable scaffolds for RNA processing.

### 2.3 Polysaccharides: Polymers of Structure, Architecture, and Energy

Complex carbohydrates (polysaccharides) represent a crucial class of biological macromolecules whose functions arise primarily through their bulk polymer properties. While DNA, RNA, and proteins often dominate the discussion of biopolymers, polysaccharides such as cellulose (a linear polymer of glucose), chitin (a linear polymer of *N*‐acetylglucosamine), and diverse extracellular matrix (ECM) components operate through branching, cross‐linking, and network formation to create hydrogels with tunable mechanical stiffness.

Within the ECM, glycosaminoglycans (GAGs) such as hyaluronan behave as semiflexible polyelectrolytes. Their high negative charge density forces these polymer chains to adopt extended conformations to minimize electrostatic repulsion, creating a large hydrodynamic volume. This expanded structure allows GAGs to retain substantial amounts of water, generating the turgor pressure essential for tissue resilience. The mesh size and degree of entanglement within these polysaccharide networks regulate the diffusion of signaling molecules, effectively acting as a physical sieve that shapes intercellular communication [116, 117, 118, 119].

The dynamics of these networks often follow the phase transition principles established by Tanaka [19]. A classic example of volume phase transition of a proteoglycan is the condensation and rapid “decondensation” (expansion) of mucin granules upon secretion. In their condensed state, calcium ions shield the negative charges on the mucin polymers, keeping the network collapsed. When calcium is exchanged for sodium, this electrostatic shielding is lost, triggering a rapid, discontinuous swelling event that hydrates and expands the mucosal surface [120]. Noncovalent cross‐linking among polysaccharide chains further imparts shear‐thinning behavior to these matrices. Under mechanical stress, the network becomes more fluid, enabling processes such as cell migration; when the stress is removed, the matrix resolidifies to maintain tissue architecture [121]. This combination of charge‐driven swelling, reversible cross‐linking, and mechanical adaptability underscores the unique polymer physics that make polysaccharides indispensable to biological structure and function.

## 3 Polymer Dynamics in Cellular Function

The core processes of the central dogma are, at their heart, search‐and‐capture problems constrained by the physical properties of biopolymers involved. Whether a transcription factor locating its promoter, a repair complex identifying a double‐strand break, or a sensor detecting oxidative stress, the efficiency of these reactions is governed by diffusion, phase separation, and the intrinsic fluctuations of polymer chains.

The preceding sections outlined the overarching physical principles that shape the behavior of the three major classes of biopolymers, while touching only briefly on their cellular roles. In the following sections, we examine two of the most important biological functions of biopolymers in greater depth, highlighting how these physical constraints are harnessed to enable precise and robust cellular decision‐making.

### 3.1 Gene Regulation and Transcription

#### 3.1.1 Condensate Formation as a Driver of Transcriptional Bursting and Cooperativity

For decades, transcriptional activation was described as a stepwise, ordered assembly of stable protein complexes on DNA. This view was supported by evidence of direct DNA binding by transcription factors (TFs) and RNA Polymerase II (RNAPII), followed by the sequential recruitment of additional regulatory components [122, 123, 124]. Although this model captures events at individual promoters, it does not fully explain cooperative activation across multiple loci, synchronous transcriptional bursts from distinct promoters, or the remarkable speed and robustness of gene induction at super enhancers.

**FIGURE 5:**
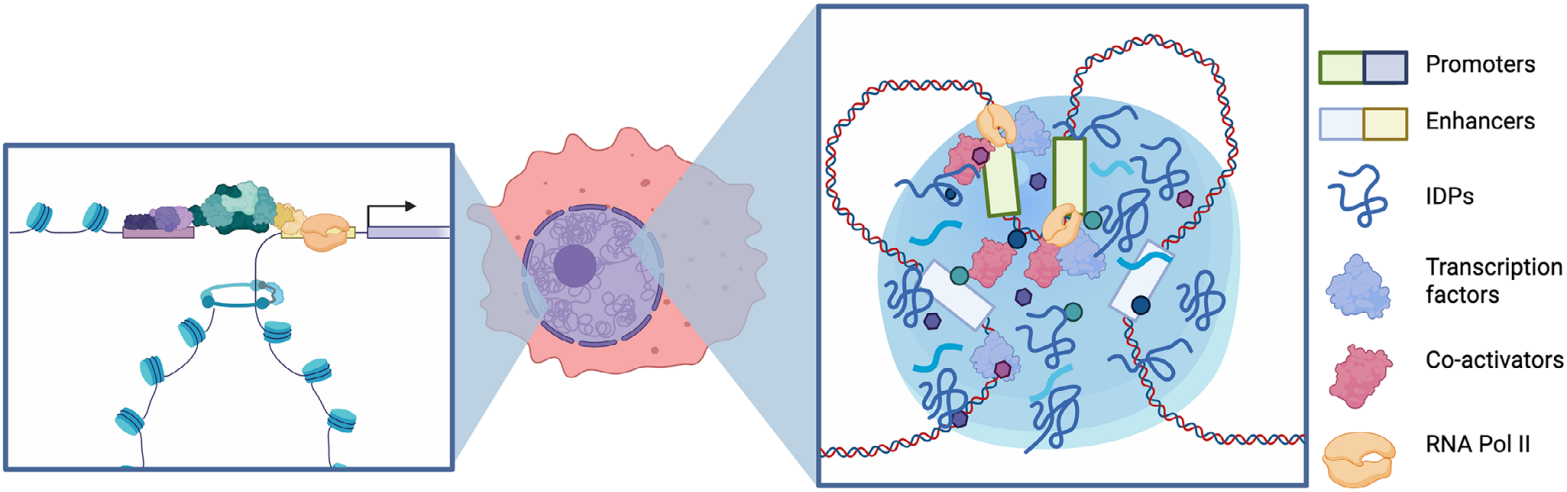
Competing models of transcriptional activation and regulation. Transcriptional regulation was proposed to be coordinated by transcriptional “hubs” formed by LLPS, which functions to recruit cofactors promoting transcription (right panel). Depicted here is one proposed mechanism where condensates drive loop formation. A competing model is via soluble transcriptional complexes (left panel). These complexes are cooperative assemblies that do not rely on a phase transition of IDPs or transcription factor IDRs and coactivators for their function. Created in BioRender: Chen, M. (2026), https://BioRender.com/tq73zeg.

Emerging evidence suggests that these phenomena arise from the collective phase behavior of the transcriptional apparatus rather than from strictly stoichiometric binding events [28, 125– 127]. At super enhancers, the high local concentration of TFs and RNAPII is achieved through LLPS, not through stable one‐to‐one interactions (Figure 5) [28, 45, 125, 128, 129]. Young, Sabari and colleagues first demonstrated that TFs and cofactors containing IDRs undergo condensation at super‐enhancers. Subsequent work showed that the unstructured C‐terminal domain (CTD) of RNAPII and the IDRs of the Mediator complex subunits co‐condense into transcriptionally active condensates [126, 127, 129, 130]. These condensates are temperature and concentration‐dependent and largely driven by the polymeric properties of the RNAPII CTD with CTD length being particularly important: from a polymer physics perspective, a longer CTD increases the number of available “stickers,” enhancing the probability of capture by early transcriptional assemblies [129].

Viewed through this lens, these transcriptional condensates act as micrometer‐scale biochemical reactors. By forming a distinct phase with a high partition coefficient for specific regulatory proteins, the cell boosts the local concentration of transcriptional components by orders of magnitude beyond what could be achieved in a well‐mixed homogeneous nucleoplasm. This framework also provides mechanistic explanation for “transcriptional bursting”—the observation that transcription occurs in intense, discontinuous pulses [128, 131]. Condensate formation is a cooperative, threshold‐dependent process: once the local concentration of TFs exceeds the *C*_sat_, a condensate droplet forms and transcription initiates rapidly within the droplet. When TF levels fall or PTMs of the transcriptional machinery—including scaffold proteins and coactivators—shift their phase boundary, the condensate dissolves, and transcription ceases. For example, FUS, an IDP that binds the CTD of RNAPII, inhibits phosphorylation of the CTD, which alters the distribution of RNAPII on genes [132]. Orthogonally, phosphorylation of FUS itself was shown to be sufficient to disrupt critical oncogenic signaling through abrogating interactions between the FUS‐CHOP oncoprotein and chromatin remodelers [77, 133].

#### 3.1.2 Chromatin Looping and the Physical Basis of Transcriptional Bursting

The dynamics of the DNA polymer itself also play a central role in regulating transcription. Enhancers reside several megabases away from their target promoters, requiring physical contact with the promoter through chromatin looping to activate transcription [44, 134]. The frequency and duration of these contacts are governed by the looping kinetics of the chromatin fiber. When chromatin is treated as a semiflexible polymer, the probability of enhancer–promoter contact is determined by parameters such as the persistence length and the excluded volume of the intervening DNA.

This raises a key question: how does the cell achieve specificity within such a stochastic system? Because the chromatin polymer undergoes constant thermal fluctuations, enhancer–promoter looping is inherently probabilistic. These fluctuations create periodic “breathing” of the polymer, generating transient temporal windows in which the loop is closed (permitting transcription) or open (preventing it). As a result, TF concentration is translated into discrete bursts of mRNA production rather than a continuous output.

In this framework, the temporal pattern of gene expression becomes a direct readout of the mechanical fluctuations of the chromatin polymer as sampled by the transcriptional machinery. The interplay between polymer physics and regulatory factor dynamics thus provides a physical basis for the stochastic yet highly regulated nature of transcriptional activation.

### 3.2 Polymer‐Driven Maintenance of Genomic Integrity

#### 3.2.1 The Search Problem

Perhaps the most daunting search task in biology is DNA repair. A typical mammalian cell must locate a single damaged base or a double‐strand break (DSB) within billions of intact base pairs—the “needle in a haystack” scenario on a genomic scale. If repair proteins relied solely on 3D diffusion to find their targets, the search time would be prohibitively long, often exceeding the cell cycle duration. Because DSB is the most catastrophic form of DNA damage and must be repaired for a cell to remain viable, evolution has optimized this process with remarkable efficiency.

To solve this high‐stakes problem, cells employ facilitated diffusion, a mechanism in which proteins reduce the dimensionality of their search. As described in the seminal theoretical work by Halford and Marko, DNA‐binding proteins do not simply diffuse freely through the nucleoplasm. Instead, they engage in transient, nonspecific interactions with the DNA backbone, allowing them to slide along the polymer in a 1D random walk [135]. This enables rapid scanning of local sequences and because DNA is a coiled polymer, proteins can also hop or jump between segments that are spatially close but genomically distant. This combination of 1D sliding (to scan local sequences) and 3D hopping (to escape local traps and cover long distances) minimizes the total search time and dramatically increases the probability of successful target acquisition.

#### 3.2.2 Repair Centers

Once a lesion is detected, the local dynamics of the chromatin polymer change dramatically. High‐resolution tracking of DSBs in yeast and mammalian cells shows that damaged loci exhibit a significant increase in mean square displacement compared to undamaged regions [136, 137, 138]. Whereas intact chromatin typically displays subdiffusive motion characteristic of a polymer embedded in a viscoelastic network, damaged sites often become more mobile and display higher diffusion coefficients. This mobilization is thought to arise from local relaxation of the chromatin fiber—potentially through nucleosome eviction, chromatin remodeling, or the changes in cross‐linking proteins.

Interestingly, while the break site itself becomes more dynamic, global chromosome mobility decreases, a phenomenon mediated by Rad51 and Rad52. The increased mobility at the DSB serves a clear functional purpose: it facilitates the search for a homology repair template. During homologous recombination, the broken DNA end must locate an identical sequence on the sister chromatid or homologous chromosome to use as a template for repair. By expanding the volume of nuclear space explored per unit time, the cell increases the probability of a productive encounter with the correct donor sequence.

**FIGURE 6:**
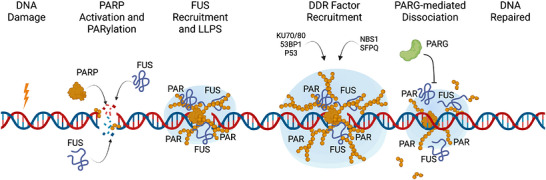
Model of the DNA damage response (DDR) in a phase‐separated repair center. Following DNA damage, an early event is PARP activation and ADP‐ribosylation or PARylation of the double strand break site. PAR accelerates FUS recruitment and phase separation around the break. DDR proteins are recruited as FUS and PAR form a growing repair center. PARG is also recruited, which functions to degrade the PAR polymer, which drives FUS dissociation following successful DNA repair. Created in BioRender: Chen, M. (2026), https://BioRender.com/tq73zeg.

Furthermore, this mobility allows multiple DSBs to cluster into “repair centers,” which are distinct phase‐separated domains rich in repair factors [88, 139, 140]. These condensates concentrate repair enzymes, coordinate signaling, and create a dedicated environment for efficient DNA repair. The ability of the DNA polymer to rearrange and coalesce into these hubs ensures that genomic integrity is restored before the cell proceeds through division (Figure 6).

## 4 Engineered Molecular Tools to Study Biopolymer Function

The elucidation of the physical principles governing biopolymers has not only deepened our understanding of cell biology but also provided a blueprint for engineering it. By treating part of the cell and cellular processes as a soft matter system, researchers can now manipulate cellular behavior through precise control of biopolymers and the polymeric environment. Synthetic condensates and biopolymer‐inspired materials can be engineered by leveraging multivalency, intrinsic disorder, and solubility to reprogram cellular processes. Below, we highlight three emerging frontiers of engineered biopolymers.

### 4.1 Synthetic Transcriptional Condensates

The spatial organization of the genome is governed by endogenous condensates during transcription. Synthetic condensates have now been developed to dissect and recreate the molecular grammar underling this process [141, 142, 143]. By characterizing the interaction dynamics of LCDs found in TFs, Chong and colleagues engineered chimeric proteins capable of nucleating droplets at specific genomic loci by recruiting endogenous coactivators and IDRs. These studies validate the stickers‐and‐spacers model of phase separation: increased coactivator concentration produces a nonlinear enhancement in transcriptional output.

Synthetic condensates can also be engineered to be mechano‐sensitive. Cai et al. demonstrated this using the mechanotransducer protein YAP [44]. By designing IDPs that phase separate only under defined osmotic or stiffness conditions, they created a mechano‐sensitive transcriptional switch that activates gene expression in response to mechanical force. This technology has clear implications for tissue engineering where scaffolds are commonly used to host stem cells for differentiation through physical cues.

Tunability of transcription remains a central engineering challenge. Chilkoti and colleagues addressed this problem using engineered elastin‐like polypeptides (ELPs) and resilin‐like polypeptides to create synthetic IDPs (synIDPs). When fused to functional protein domains, these synIDPs can sequester target DNA and amplify transcription in both bacteria and mammalian cells, enabling thermal and temporal control over gene expression [143].

### 4.2 Synthetic Translational Condensates

While transcriptional condensates regulate mRNA production, synthetic translational condensates extend this control out of the nucleus and into the cytoplasm to modulate protein synthesis. Multiple groups have engineered condensates that recruit specific mRNAs along with the translational machinery required to process them [144, 145, 146]. By designing synthetic organelles that admit only specific tRNAs or ribosomes, researchers can achieve highly orthogonal translation systems.

While some approaches rely on genome engineering, others exploit the spatial control afforded by phase separating proteins. Synthetic condensates to control translation have been created to interface with endogenous cellular pathways. For example, polypeptide tags have been developed to drive phase separation of native enzymes and boost catalytic activity [145]. Demonstrating this concept, Chilkoti and colleagues engineered artificial IDPs (A‐IDPs) based off native resilin to form intracellular condensates capable of modulating enzymatic reactions [147]. A‐IDPs recruited B‐galactosidase into phase‐separated droplets resulting in increased catalytic efficiency of the enzyme. This enhancement was also tunable and increasing the molecular weight of the A‐IDP led to more efficient substrate sequestration.

mRNA sequestration through phase separation is another strategy to regulate expression of a target protein [146]. Interestingly, the impact of mRNA sequestration has the opposite effects in protocells (reduced translation) vs. *Escherichia coli* (enhanced translation), likely reflecting differences in ribosome dynamics in vitro and in vivo. This contrast underscores the need to understand both polymer physics and biological context in which engineered operate. Inspired by stress granules, researchers have also engineered IDPs that reversibly arrest translation in response to chemical or environmental cues (such as light), effectively creating a molecular “pause button” for cellular processes [148, 149].

### 4.3 Secreted IDPs

Nature uses secreted biopolymers such as elastin, mucin, and silk to create functional materials important for the organism's survival; some of these such as tropoelastin—the precursor of cross‐linked elastin fibers—are themselves IDPs. Elastin‐like polypeptides are a class of SynIDP inspired by tropoelastin. ELPs are composed of repetitive amino acid sequences derived from human tropoelastin and exhibit lower critical solution temperature (LCST) behavior like tropoelastin. Below their cloud‐point temperature, ELPs are soluble and above it, they transition into an insoluble coacervate phase. ELPs and their derivatives have been designed for numerous biomedical application: (1) purification of proteins and viruses [150, 151, 152]; (2) delivery of small molecule drugs [153, 154, 155]; and (3) injectable depots for delivery of biologics [156, 157, 158, 159].

Next generation SynIDPs have also been designed by Chilkoti and colleagues that go beyond the spacer only design of ELPs and begin to recapitulate the sticker and spacer design of native IDP with periodic copies of a weak hydrophobic sticker—Ala_25_—embedded in a disordered ELP. These SynIDPs—termed partially ordered polymers (POPs)—undergo an LCST phase transition like ELPs [160, 161]. Unlike ELPS that undergoes LLPS with complete thermal reversibility with no hysteresis, POPs exhibit tunable thermal hysteresis in their phase behavior, the magnitude of which is proportional to the fraction of the oligoalanine spacer in the sequence, and form highly porous solids that are fractal gels upon undergoing their phase transition. Because they can be designed at the sequence level to be a liquid at room temperature and a porous solid at physiologic temperature, POPs are under active development as injectable scaffolds for tissue regeneration [162].

PTMs present another powerful strategy to diversify the structural and phase behavior of ELPs beyond solely the amino acid sequence. One approach is myristoylation of ELPs through coexpression of *N*‐myristoyltransferase in *E. coli*, which enables for the site‐specific attachment of myristic acid [163]. This modification preserves the inherent LCST behavior of the ELP while providing a hydrophobic core that drives self‐assembly with genetically programmable features like size and shape. These fatty‐acid modified ELPs (FAMEs) combine lipid hierarchical assembly with the thermoresponsiveness of the ELP to create hybrid materials with emergent behaviors. Recent biophysical studies also demonstrate that lipidation alters the protein's hydration and backbone structure, directly influencing the transition temperature and nanomorphology of the resulting assemblies [164, 165]. The conjugation of other PTMs—including cholesterol (cholesterol‐modified polypeptides, or CHaMPs [166]) and broader sterols (sterol‐modified polypeptides, or STaMPs [167]—can also create materials with diverse hierarchical structures including spherical and cylindrical micelles, and vesicles. These PTMs predictably shift the LCST cloud point according to the hydrophobicity of the lipid, offering a method to engineer controllable phase behavior and structural hierarchy. Peptide–polymer hybrids can also be conjugated to biological peptides that self‐assemble into fibers or hydrogels in the extracellular space, mimicking ECM architecture [168, 169, 170]. Together these technologies represent the ultimate convergence of polymer concepts and synthetic biology.

## 5 Biophysical Approaches for Studying Biopolymers

**FIGURE 7:**
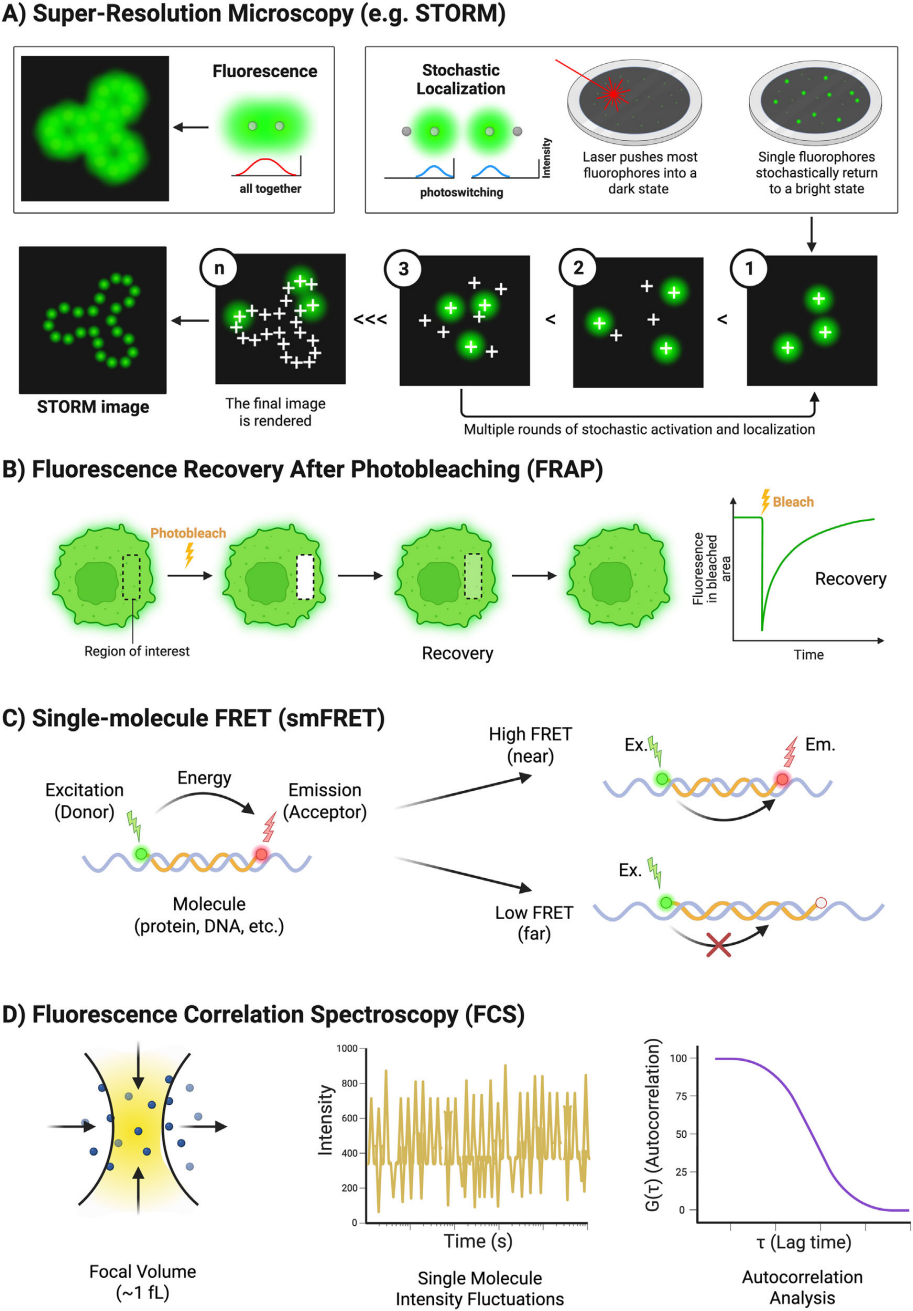
Fundamental principles underlying key biophysical techniques. (A) Super‐resolution microscopy (e.g., STORM/PALM). Exceeds the limit of diffraction in conventional microscopy through the principle of stochastic activation and localization of individual fluorophores that can be reconstructed into a super‐resolution image. (B) Fluorescence recovery after photobleaching (FRAP). Measures bulk diffusion and mobile fractions through cycles of prebleach, bleaching, and recovery using a high‐intensity laser. (C) Single‐molecule FRET (smFRET). Uses the principle of distance‐dependent energy transfer between excited donor and acceptor fluorophores. Intensity spikes measure energy transfer. (D) Fluorescence correlation spectroscopy (FCS). Measures single‐molecule dynamics and concentrations by detecting changes in fluorescence intensity through a focal volume. Created in BioRender: Chen, M. (2026), https://BioRender.com/tq73zeg.

Advances in microscopy and biophysical instrumentation over the last two decades have enabled direct empirical validation of theoretical predictions about biopolymer behavior. These tools overcome the challenges posed by nanoscale polymer dynamics that unfold over millisecond timescales. Techniques such as super‐resolution microscopy, dynamic photobleaching assays, single‐molecule Förster resonance energy transfer (smFRET), and fluorescence correlation spectroscopy (FCS) now allow researchers to visualize conformational fluctuations and quantify the physical properties of individual biopolymer molecules (Figure 7).

### 5.1 Super‐Resolution Microscopy

Conventional optical microscopy is constrained by the diffraction limit of light, which prevents resolution of features smaller than roughly 250 nm. For many biopolymers, this limitation obscures critical structural details, causing polymer networks—such as actin microfilaments or chromatin—to appear as amorphous blobs rather than discrete, entangled chains.

Super‐resolution microscopy techniques, such as stochastic optical reconstruction microscopy (STORM) and photoactivated localization microscopy (PALM), circumvent this barrier and achieve resolutions of approximately 20 nm [171, 172]. These methods rely on the temporal separation of fluorescent emitters to break the diffraction barrier limit of light; by stochastically switching individual fluorophores between “on” and “off” states, researchers can localize the center of each point spread function with high precision (Figure 7A).

Using these approaches, structures once thought to be continuous bundles—such as actin filaments—were revealed to be discrete, cross‐linked fibers. STORM has also been used to visualize chromatin loop “clutches” within the nucleus [173]. Integrating emerging molecular tools such as VECTOR and synthetic condensates with super‐resolution microscopy techniques will further illuminate poorly understood biological phenomena, including long‐range enhancer–promoter contacts and the relationship between the organization of biopolymers and their simulations [130, 174]. Continued development of noninvasive, high‐speed imaging methods capable of resolving ∼50 nm features in living cells will be essential for observing dynamic polymer behavior in real time [175, 176]. Super‐resolution microscopy enables detailed visualization and analysis of the spatial organization and architecture of biopolymers, offering the highest spatial resolution among currently available imaging techniques.

### 5.2 Dynamic Photobleaching Assays

While super‐resolution microscopy provides spatial and structural information, understanding the physical state of a biopolymer also requires measuring its mobility. Fluorescence recovery after photobleaching (FRAP) is one of the most widely used methods to quantify diffusion coefficients and the mobile fraction of molecules within condensates [177, 178]. FRAP offers a means to estimate viscosity and mobility of a biopolymer in the cell, provided its limitations are carefully considered.

Standard FRAP analyses assume steady‐state conditions and pure Brownian diffusion, yet recovery in biological systems is often influenced by binding interactions in addition to polymer viscosity [179]. In a typical FRAP experiment, a high‐intensity laser photobleaches a specific region of interest containing fluorescently labeled molecules (Figure 7B). Recovery of fluorescence depends on the diffusion of unbleached molecules into the bleached zone, enabling estimation of diffusion coefficients, mobile fractions, and—when combined with reaction–diffusion modeling—binding residence times [180]. However, photobleaching itself can introduce artifacts, including local heating and free‐radical generation [181, 182].

FRAP was instrumental in demonstrating LLPS in P‐granules that were photobleached and shown to recover rapidly within seconds, indicating that their constituent proteins and RNA are highly mobile [85]. In contrast, solid protein aggregates, such as amyloid fibrils, show negligible recovery after photobleaching, reflecting their rigid, immobile structure. Compared with super‐resolution microscopy, FRAP sacrifices spatial detail but provides kinetic insight by measuring bulk diffusion as a proxy for condensate fluidity. Together, the two techniques are highly complementary: super‐resolution microscopy maps the spatial organization of biopolymers, while FRAP quantifies their dynamic exchange. When paired, they enable visualization and measurement of how mobile biopolymer clients move, interact, and become transiently trapped across cellular compartments.

### 5.3 Single‐Molecule FRET (smFRET)

FRET enables measurement of distances from 1 to 10 nm between biomolecules or between regions of the same molecule [183, 184, 185, 186, 187]. A donor fluorophore is used to label one molecule (or part of a molecule) of interest and a second acceptor fluorophore is used to label a second molecule or a different region of the same molecule (Figure 7C). When the donor is excited by a laser, it transfers energy nonradiatively to the acceptor when the two are within range, with transfer efficiency following an inverse sixth‐power dependence on distance. This steep distance dependence makes FRET exquisitely sensitive to small conformational changes.

Traditional “bulk” FRET averages signal from millions of molecules, making it useful for studying protein–protein interactions or designing biosensors, but is unable to resolve molecular heterogeneity. smFRET overcomes this limitation by operating at picomolar concentrations, allowing detection of individual molecules, and generating histograms of FRET efficiencies that reveal distinct conformational states [185, 188]. These histograms may show a single peak, multiple peaks, or a broad distribution, each reflecting different structural or dynamic properties [189].

smFRET therefore produces conformational landscapes of molecules and is a powerful method to study polymer physics. For example, smFRET was used to empirically demonstrate that IDPs follow standard polymer scaling laws by determining the dimensions of chain segments with different lengths using unfolded proteins, foldable proteins, and highly charged IDPs in different chemical environments [190, 191]. Another application of smFRET was to study the effect of charge interactions on IDP dimensions. Particularly relevant to the stickers‐and‐spacers model, smFRET was used to show that IDPs behave as charged polymers [80]. smFRET enabled isolation of only unfolded molecules to measure their radius of gyration under low salt and high salt conditions. These experiments showed that net charge on the IDP drives expansion behaving like a repulsive spring, and that charge balance drives collapse.

### 5.4 Fluorescence Correlation Spectroscopy (FCS)

Another technique with single molecule sensitivity is fluorescence correlation spectroscopy, or FCS [192, 193, 194]. FCS uses a focused laser to create a femtoliter‐scale “focal volume” and detects fluorescence fluctuations as molecules stochastically enter and exit this illuminated region. These intensity fluctuations are analyzed by autocorrelation to extract a diffusion coefficient with nanomolar or better sensitivity (Figure 7D). Beyond diffusion, FCS can quantify the local concentration of biomolecules, their binding interactions and hydrodynamic radius, enabling real‐time measurements at concentrations down to picomolar range.

The comparative strength of FCS over FRAP lies in its ability to quantify single‐molecule dynamics rather than bulk exchange kinetics. Traditional single‐color FCS relies on a change in mobility of the molecule to infer molecular interactions. This limitation has been addressed by variants such as dual‐color fluorescence cross‐correlation spectroscopy, which enables direct observation of two distinct molecules diffusing as a single complex [195]. Additional approaches, such as scanning FCS or FRET‐based methods further extend the capabilities of FCS to systems characterized by low mobility [196].

A recent study by Galvanetto et al. demonstrated the power of FCS to probe condensate dynamics at the molecular scale. By integrating smFRET, nanosecond FCS (nsFCS), and molecular dynamics simulations, they quantified intramolecular distance distributions and dynamics on the nanosecond timescale [197]. Their results revealed that despite the macroscopic viscosity of condensates, local side‐chain interactions are highly dynamic at the molecular length scale, which explains how biochemical reactions occur efficiently within condensates [131].

Biophysical studies increasingly rely on techniques such as FCS to obtain quantitative measurements that go beyond qualitative mesoscale observations. The spatiotemporal resolution of FCS can be further enhanced by combining it with super‐resolution imaging. For a focused discussion of the capabilities, limitations, and future directions of FCS, an excellent review by Sankaran and Wohland provides deeper insight [198].

## 6 Emerging Concepts and Perspectives

Despite the transformative success of integrating polymer concepts into cell biology, we remain at the beginning of understanding how physical principles shape the complexity of living systems. As biophysical approaches deepen our mechanistic and quantitative understanding of cellular processes, they also reveal the limitations of classical polymer models. Living cells are fundamentally nonequilibrium systems with constant energy input through metabolism. The continual input and consumption of energy is required to perform biological reactions, maintain ion gradients, and synthesize the cellular architecture to support life. The quantitative precision required to fully model or engineer cellular systems is still lacking, and the full transition from qualitative descriptions of biology to predictive engineering is currently obstructed by significant theoretical and technical bottlenecks. Here we explore a few important emerging concepts and open challenges necessary to solve to achieve this goal.

### 6.1 Active Soft Matter

The conformational fluctuations of a polymer chain are driven by thermal collisions with solvent molecules in passive polymer solutions. However, the cellular interior is populated by a mixture of both passive components and active components [199]. For example, molecular motors such as myosin walking on actin, kinesin and dynein traversing microtubules, and RNA polymerases sliding along DNA are active components requiring energy input. These enzymes hydrolyze ATP to exert directed forces on the polymer network, injecting mechanical energy into the system. These active components that are driven by internal energy consumption are termed active matter. While this terminology may seem trivial, studying active matter is not trivial, and creative biophysical techniques are essential to a physical understanding of biology. An example is using single‐walled carbon nanotubes (SWNTs) to track proteins and monitor intracellular dynamics [200]. SWNTs are highly stable, nonbleaching probes that were used to map intracellular fluctuations across a broad range of timescales. Using SWNTs, it was shown that at short timescales, the cytoplasm behaves like a passive thermal bath, but at longer timescales, the fluctuations are dominated by active motor forces, exceeding thermal predictions by orders of magnitude. In effect, these forces create a “superthermal” environment essential for mixing reactants in the viscous cytosol and for the rapid remodeling of the cytoskeletal polymer network during cell migration and division. This example highlights the importance of quantitative measurements and studying the timescales of biological reactions that is enabled only by biophysical techniques.

### 6.2 Arrested Biology

At the opposite extreme from active matter lies arrested biology. While active matter describes the nonequilibrium nature of cells, recent work shows that the cytoplasm can also transition into a solid‐like glassy state. A glass transition occurs when a liquid is cooled or compressed to the point where molecular crowding becomes so extreme that particles are trapped in “cages” formed by their neighboring molecules. The molecules can vibrate in place, but they cannot diffuse long distances; the material retains the disordered structure of a liquid but the mechanical rigidity of a solid, like glass. A landmark study demonstrated that bacteria can exploit this physical transition as a survival strategy [201]. By tracking the motion of protein aggregates and distinct genetic loci in *Caulobacter crescentus* and *E. coli*, Parry and colleagues observed that as cellular metabolism slowed due to nutrient deprivation or ATP depletion, the cytoplasm underwent a sharp transition from a fluid to a solid. The cytoplasm became arrested, freezing diffusive transport and biochemical reactions. This transition allows the cell to enter a state of dormancy, effectively pausing its biological clock. Remarkably, this transition is reversible: when nutrients are restored, the cytoplasm transitions back into a fluid, and metabolism resumes. Similar behavior has also been observed in yeast, regulated by both nutrients and pH [23, 202]. Arrested biology provides a physical explanation for the remarkable resilience of bacterial spores, seeds, and extremophiles like tardigrades. Together with active matter, these concepts reveal that the cell is a material with tunable physical properties.

### 6.3 Predictive Polymer Behavior

Despite major advances, we still cannot predict the material properties and behavior of a cell from its genomic or proteomic composition. While LLPS can be described using Flory–Huggins theory, we cannot yet compute the phase diagram of a living cell. In polymer chemistry, chain length, charge density, and solvent conditions provide reasonable approximations of the binodal curve. In biology, this predictability collapses due to the immense complexity of the intracellular environment. The cytoplasm is not a binary mixture, but a multicomponent soup of thousands of interacting species. Current models reduce this complexity to binary or ternary system (e.g., protein + RNA + buffer), which oversimplifies the competitive interactions in vivo. PTMs further complicate prediction by dynamically altering charge distribution and hydrophobicity of IDPs. A single phosphorylation event can shift the *C*_sat_ by orders of magnitude, dissolving a functional condensate or promoting pathological aggregation. In this sense, we lack a “physical code” analogous to the genetic code.

The molecular grammar of IDPs is beginning to be deciphered from recent studies [82, 156, 203, 204, 205, 206]. Once fully deciphered, it may allow us to read a protein sequence and predict: (1) whether it will phase separate, (2) under what conditions, and (3) the material properties—such as the storage and loss modulus, viscosity, and surface tension—of the resulting condensate. Achieving this goal will require high‐throughput platforms to map phase diagrams of complex mixtures and the integration of machine learning and large language models to identify the sequence motifs that govern phase behavior in crowded cellular environments. The Hyman lab and others initially defined the “stickers‐and‐spacers” framework underlying the driving forces of phase separation of FUS and similar RNA‐binding proteins as the first steps toward achieving this goal [82, 207]. Since then, additional methods have been created for predicting disorder in protein sequences and intermolecular interactions between IDRs by Holehouse and colleagues [208, 209, 210]. More recently, multimodal generative deep learning tools were created by the Holehouse lab to discover sequence‐ensemble relationships of IDPs [211].

Particularly important to these computational approaches are experimental validation strategies performed in parallel with computational cross‐validation. The specific techniques used will vary but commonly include small‐angle X‐ray scattering (SAXS), dynamic light scattering, NMR, FRET, and other methods discussed in this review. If deep learning approaches can accurately predict experimental results such as SAXS profiles, then they would encapsulate true understanding of the interplay between IDP sequence and biophysical properties, enabling library‐scale design of IDPs/IDRs and accelerating the experimental testing of sequence–ensemble–function hypotheses.

### 6.4 Function vs. Epiphenomenon

As cell biology has become increasingly intertwined with physical biology, it also confronted a central question: are the polymer‐like behaviors we observe functional mechanisms or incidental physical consequences? This debate is most prominent in the context of transcriptional condensates. The prevailing hypothesis proposes that LLPS is a regulatory mechanism that concentrates transcription factors to enhance reaction rates and driving robust gene expression [45, 131, 142, 212, 213, 214]. Similar models have been proposed for cellular processes ranging from DNA repair to membrane signaling [28, 101, 131, 140, 215]. In this view, phase separation is a driver rather than a passenger.

However, a growing body of work challenges this interpretation. McSwiggen et al. used stroboscopic photoactivatable single particle tracking (spaSPT) to examine RNAPII dynamics in HSV‐infected cells [91, 216]. They discovered that while RNAPII was concentrated in viral replication compartments (RCs), this was due to RNAPII binding interactions with DNA and other RNAPII molecules rather than LLPS as evidenced by the unrestricted diffusion of RNAPII across RC boundaries by spaSPT. The authors concluded that what appears to be a phase‐separated droplet may instead be a cluster of specific molecular interactions lacking emergent liquid‐like properties such as surface tension or phase behavior. In this alternative view, LLPS is an epiphenomenon: proteins containing IDRs are capable of phase separation, but this physical property may not be the mechanism regulating transcription.

More recently, Bremer et al. addressed this question in a yeast model [94]. Using engineered variants of yeast transcription factor Gcn4, they found that DNA acts not as a nucleating scaffold, but rather a dissolver of transcriptional condensates. Transcriptional output correlated with binding affinity to Med15, not with condensate formation. In fact, condensates acted more as a trap for Gcn4, which can function as a soluble complex without requiring condensation. One challenge to answering this question is the overlapping interactions that drive both phase separation and formation of soluble complexes as mentioned by the authors. This question of causality remains unresolved, but it remains the most important question to answer in this field. Its resolution will shape both basic research and translational efforts aimed at harnessing physical processes in living systems [92, 217].

## 7 Conclusions

Looking ahead, the next decade of research will likely be defined by the early translation of physical principles into new molecular tools for discovery and therapeutic intervention. The term “condensatopathies” refers to diseases driven by aberrant phase transitions, and it is rapidly emerging as a central theme in biology. This emergence is most evident in neurodegenerative disorders such as amyotrophic lateral sclerosis, Huntington's, and Alzheimer's disease [218, 219, 220, 221]. Increasing evidence suggests that the pathological aggregates observed in these conditions (e.g., FUS, TDP‐43, and Tau inclusions) often begin as functional liquid droplets that undergo an irreversible phase transition into solid fibrils. In the context of this review, however, the term “condensatopathy” may be too narrow a term as it captures only one facet of polymer behavior in living systems. Future therapeutics will likely move beyond inhibiting specific binding sites toward strategies that modulate the material state of these polymers of life. The goal will be to engineer both molecules and eventually devices that can alter the physical properties of cells and even whole organisms.

At the same time, the convergence of polymer physics and biology is poised to drive the bottom‐up engineering of life. Advances in active soft matter will enable the integration of molecular motors into synthetic polymer networks, bringing researchers closer to constructing “synthetic cells” capable of autonomous movement, replication, and adaptation. As we uncover, formalize, and ultimately master the rules governing nonequilibrium thermodynamics and active matter in polymer networks, we move toward a future in which we do not merely observe the physics of life, but begin to control it.

## Conflicts of Interest

The authors declare no conflicts of interest.

## Data Availability

Data sharing not applicable to this article as no datasets were generated or analyzed during the current study.
